# A systematic review and meta-analysis of bovine brucellosis seroprevalence in Latin America and the Caribbean

**DOI:** 10.1016/j.nmni.2023.101168

**Published:** 2023-08-24

**Authors:** D. Katterine Bonilla-Aldana, Adrián Esteban Trejos-Mendoza, Soffia Pérez-Vargas, Estefany Rivera-Casas, Fausto Muñoz-Lara, Lysien I. Zambrano, Kovy Arteaga-Livias, Juan R. Ulloque-Badaracco, Esteban A. Alarcon-Braga, Enrique A. Hernandez-Bustamante, Ali Al-kassab-Córdova, Vicente A. Benites-Zapata, Alfonso J. Rodriguez-Morales

**Affiliations:** a Research Unit, Universidad Continental, Huancayo, Peru; b Faculty of Veterinary Medicine, Fundación Universitaria Autónoma de las Américas-Institución Universitaria Visión de las Américas, Pereira, Risaralda, Colombia; c Department of Internal Medicine, Facultad de Ciencias Médicas (FCM), Universidad Nacional Autónoma de Honduras (UNAH), Tegucigalpa DC, Honduras; d Department of Internal Medicine, Hospital Escuela, Tegucigalpa, Honduras; e Department of Morphological Sciences, School of Medical, Sciences, Universidad Nacional Autónoma de Honduras, Tegucigalpa, Honduras; f Facultad de Medicina, Universidad Nacional Hermilio Valdizán, Huánuco, Peru; g Escuela de Medicina, Filial Ica, Universidad Privada San Juan Bautista, Ica, Peru; h Master in Clinical Epidemiology and Biostatistics, Universidad Científica del Sur, Lima, Peru; i Facultad de Ciencias de la Saud, Universidad Peruana de Ciencias Aplicadas, Lima, Peru; j Grupo Peruano de Investigación Epidemiológica, Unidad para la Generación y Síntesis de Evidencias en Salud, Universidad San Ignacio de Loyola, Lima, Peru; k Unidad de Investigación para la Generación y Síntesis de Evidencias en Salud, Vicerrectorado de Investigación, Universidad San Ignacio de Loyola, Lima, Peru; l Centro de Excelencia en Investigaciones Económicas y Sociales en Salud, Universidad San Ignacio de Loyola, Lima, Peru; m Grupo de Investigación Biomedicina, Faculty of Medicine, Fundación Universitaria Autónoma de las Américas-Institución Universitaria Visión de las Américas, Pereira, Risaralda, Colombia; n Gilbert and Rose-Marie Chagoury School of Medicine, Lebanese American University, Beirut P.O. Box 36, Lebanon

**Keywords:** Cattle, Brucellosis, Systematic review, Meta-analysis, Seroprevalence, Zoonotic

## Abstract

**Introduction:**

Bovine brucellosis is a significant public health problem in countries with economic and zoonotic implications. Although relevant, there are no previous systematic reviews about bovine brucellosis in Latin America.

**Methods:**

We performed a systematic literature review in five data-bases to assess the seroprevalence of Brucella in cattle. A meta-analysis with a random-effects model was performed to calculate the pooled prevalence and 95% confidence intervals (95%CI). In addition, measures of heterogeneity (Cochran's Q statistic and I^2^ test) were reported.

**Results:**

The literature search yielded 3,403 articles, of which 65 studies were fully valid for analysis. The pooled seroprevalence for Brucella in bovine (n ​= ​46,883,177) was 4.0% (95%CI: 3.0%–5.0%; p ​< ​0.001), and Venezuela was the country with the highest prevalence (16.0%). By regions, the highest seroprevalence is in Central America and the Caribbean islands (8.0%,95%CI: 3.0%–15.0%; p ​< ​0.001, I^2^ ​= ​99.85).

**Conclusions:**

Some countries reported still relevant seroprevalences of bovine brucellosis, especially at the Central America and Caribbean islands. Multiple factors may influence the survival and spread of pathogens in the environment; farms located in regions bordering forest areas, in areas of difficult access to the veterinary service; extensive beef herds raised at pastures with different age and productive groups inter-mingled, and minimal concerns regarding hygiene practices and disease prevention measures. Bovine brucellosis has not been eliminated and needs to be considered with new tools for prevention and control, especially being a zoonosis.

## 1 Introduction

Bovine brucellosis is still a significant public health problem in different countries, with multiple economic and zoonotic implications [[1], [2], [3]]. This zoonosis is responsible for substantial reproductive disorders and production losses in cattle [4]. Surveillance and control are regulated by multiple countries with specific conditions to obtain and maintain the official free status, which facilitates access to export markets.

Although the World Health Organization (WHO) and the World Organization for Animal Health (WOAH) have recommended strategies and measures to either control or eradicate brucellosis, only some developed countries have achieved being free of animal brucellosis [5]. In developing countries, e.g., most Latin American countries, brucellosis remains a severe problem, and the importance of its control has attracted little attention [[1], [2], [3], [4], [5]]. Furthermore, the strategies and measures are challenging to implement effectively in countries with limited resources because eliminating brucellosis is expensive, time-consuming, and labour-intensive. Therefore, more effort is needed to explore the appropriate strategies and measures in developing countries [5].

Although relevant, there are no previous systematic reviews and meta-analyses on bovine brucellosis in Latin America. Hence, we aimed to estimate the combined seroprevalence of brucellosis in bovines from Latin America.

## 2 Methods

This systematic review was conducted following the guidelines of the Preferred Reporting Items for Systematic Reviews and Meta-analysis (PRISMA) [6] and the Cochrane Handbook of Systematic Reviews [7]. The PRISMA 2020 checklist was used throughout the review process (Supplementary Table S1).

### 2.1 Information sources and search strategy

We carried out a systematic review using the following databases: PubMed, Web of Sciences, Embase, Scopus, and LILACS, through the following search terms: "bovine," "brucellosis," and "Brucella".This search was done on October 14, 2022. No language and time filters were applied during the search. The complete search strategy can be found in Supplementary Table S2.

### 2.2 Eligibility criteria

Published peer-reviewed articles reported infection in bovine with a serological diagnosis of brucellosis were included. Diagnosis of brucellosis was made from blood samples to perform serological tests. Not milk samples were considered. For serological tests, we considered the Rose Bengal test (RBT), 2- mercaptoethanol test (2MET), enzyme-linked immunosorbent assay (ELISA), indirect immunofluorescence test (IFI), or immunofluorescence antibody test (IFAT), with the limitations in their relative differences in sensitivity and specificity. Also, the following article types were excluded: Review articles, opinion articles, and letters not presenting original data.

### 2.3 Study selection

The results of the initial search strategy were uploaded to the data management software Rayyan QCRI. Four reviewers (DKB-A, JRU-B, EAA-B and EAH-B) independently screened the title and abstract of each study. After that, the full texts of the selected articles were examined independently, deeming the selection criteria by four reviewers (AET-M, SP-V, ER-C, and FM-L). Observational studies that reported the frequency of bovine infected by *Brucella* spp. were included for quantitative synthesis. The discrepancies over the inclusion/exclusion of an article were discussed among the researchers, and a consensus was reached in all cases.

### 2.4 Data collection process and data items

Four reviewers collected the following information independently (LI-Z, KA-L, A.A-C and VB-Z) in a Microsoft Excel Sheet: type of publication, the publishing institution, country, year, and date of publication, as well as the number of infected animals assessed by serological tests.

### 2.5 Risk of bias assessment for cross-sectional studies

The risk of bias in the included studies was evaluated through the Critical Appraisal Checklist for prevalence studies proposed by The Joanna Briggs Institute (JBI) [8]. This tool has nine items and categorized each item with “yes”, “no”, “unclear” and “not applicable" and a star was given for each "yes".A study with seven or more stars was considered a low risk of bias. Meanwhile, a study with less than seven stars was considered a high risk of bias. Four reviewers (AJR-M, VB-Z, JRU-B and DKB-A) independently analysed the included studies. In case of disagreements, they were resolved by consensus.

### 2.6 Publication bias

As stated in previous studies, both Funnel Plots and Egger's Test are inaccurate for assessing publication bias in proportional meta-analysis. These tests were created assuming that studies with positive results were published more frequently than those with negative results. However, in prevalence studies, there is no consensus on what is a positive or negative result [9,10]. Due to all this explanation, the evaluation of publication bias was not carried out.

### 2.7 Data analysis

The data analysis was performed in STATA 16.0 (Stata Corporation, College Station, TX, USA). The pooled analysis assessed the rates of bovine infection by *Brucella* spp. A random effects model (Dersimonian and Laird method) was used for the meta-analysis. The 95% confidence intervals (95%CI) were calculated using the Clopper-Pearson Method. Moreover, the I^2^ statistic and Cochran Q tests were used to evaluate the heterogeneity between studies. Values greater than 60% were a sign of severe heterogeneity for the I^2^ statistic, and a p-value <0.05 was a sign of heterogeneity for the Cochran Q test. Subgroup analyses were performed according to the bovine species, countries, and geographical regions. Also, sensitivity analysis was conducted, excluding studies with a high risk of bias.

## 3 Results

### 3.1 Study selection and characteristics

**Fig. 1:**
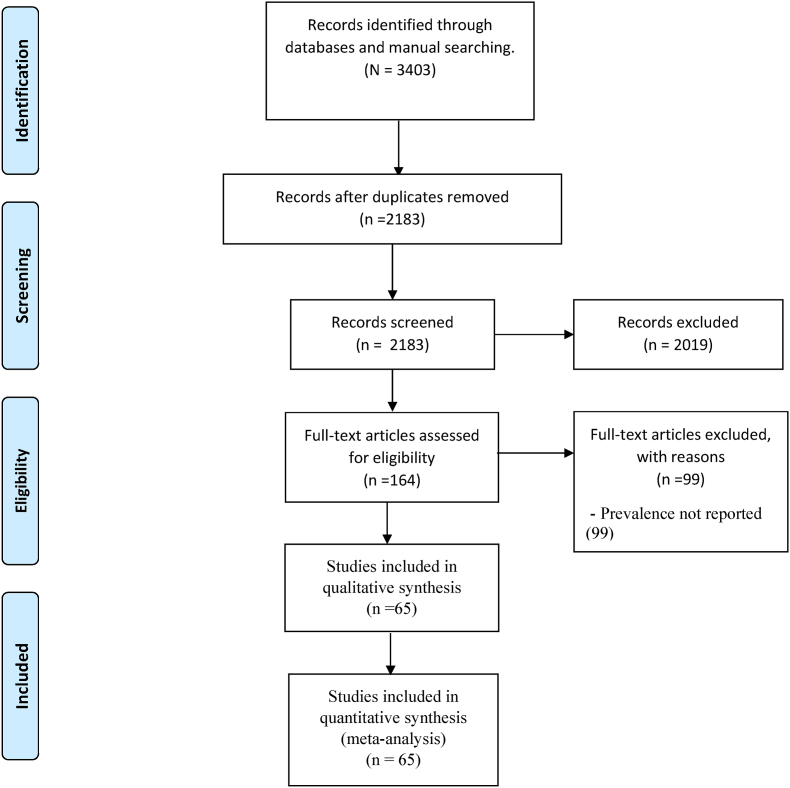
Prisma flow diagram.

A total of 3,403 articles were retrieved by the search strategy, of which 1,220 were duplicates, resulting in 2,183 records. After screening by abstract and title, 164 papers were selected for full-text assessment. Of these, 99 were excluded, and 65 studies [[11], [12], [13], [14], [15], [16], [17], [18], [19], [20], [21], [22], [23], [24], [25], [26], [27], [28], [29], [30], [31], [32], [33], [34], [35], [36], [37], [38], [39], [40], [41], [42], [43], [44], [45], [46], [47], [48], [49], [50], [51], [52], [53], [54], [55], [56], [57], [58], [59], [60], [61], [62], [63], [64], [65], [66], [67], [68], [69], [70], [71], [72], [73], [74], [75]] were finally included for the final qualitative and quantitative meta-analysis (Fig. 1). The main characteristics of the included studies are shown in Table 1.

**Table 1:** Characteristics of the included studies on bovine brucellosis in Latin America and the Caribbean.

Author	Year of publication	Country	Animals evaluated (common-name)	Number of samples tested	Number of samples positive	Prevalence	Test	Reference
*Zimmer* P et al.	2010	Argentina	Buffaloes	462	187	40.48	RBT	[22]
*Konrad* J et al.	2013	Argentina	Buffaloes	500	33	6.60	RBT	[66]
*Aznar* M et al.	2015	Argentina	Cattle	8,965	157	1.75	IFI	[42]
*Alves* A et al.	2009	Brazil	Cattle	10,803	81	0.75	ELISA	[12]
*Azevedo* S et,al	2009	Brazil	Cattle	5,351	88	1.64	RBT	[16]
*Chate* S et al.	2009	Brazil	Cattle	9,466	727	7.68	RBT	[24]
*Gonçalves* V et al.	2009	Brazil	Cattle	2,019	7	0.35	RBT	[35]
*Klein-Gunnewiek M* et al.	2009	Brazil	Cattle	8,239	248	3.01	RBT	[36]
*Marvulo M* et al.	2009	Brazil	Cattle	16,072	111	0.69	2 ​ME	[38]
*Ogata S* et al.	2009	Brazil	Cattle	20,908	688	3.29	RBT	[40]
*Rocha W* et al.	2009	Brazil	Cattle	10,738	240	2.24	RBT	[44]
*Negreiros R* et al.	2009	Brazil	Cattle	13,684	1396	10.20	RBT	[50]
*Silva V* et al.	2009	Brazil	Cattle	4,640	134	2.89	RBT	[51]
*Villar K* et al.	2009	Brazil	Cattle	9,703	560	5.77	ELISA	[52]
*Chaves NP* et al.	2012	Brazil	Buffaloes	232	12	5.17	RBT	[25]
*Borba M* et al.	2013	Brazil	Cattle	6,779	112	1.65	ELISA	[11]
*Oliveira R* et al.	2013	Brazil	Cattle	771	15	1.95	RBT	[67]
*Da Silva J* et al.	2014	Brazil	Buffaloes	4,796	176	3.67	RBT	[70]
*De Lima A* et al.	2015	Brazil	Buffaloes	136	2	1.47	IFAT	[19]
*Anzai EK* et al.	2016	Brazil	Cattle	5,067	83	1.64	RBT	[14]
*Barddal J* et al.	2016	Brazil	Cattle	12,435	464	3.73	RBT	[17]
*Baumgarten K* et al.	2016	Brazil	Cattle	1,653	11	0.67	RBT	[18]
*Almeida E* et al.	2016	Brazil	Cattle	3,901	52	1.33	ELISA	[28]
*De Oliveira* L et al.	2016	Brazil	Cattle	18,990	113	0.60	RBT	[29]
*Barbosa* J et al.	2016	Brazil	Buffaloes	5,163	443	8.58	RBT	[31]
*Dias RA* et al.	2016	Brazil	Cattle	12,920	227	1.76	RBT	[32]
*Dos Santos* L et al.	2017	Brazil	Buffaloes	390	21	5.38	RBT	[30]
*Mioni M* et al.	2018	Brazil	Cattle	1,490	33	2.21	RBT	[21]
*Carrillo H* et al.	2019	Brazil	Cattle	1,800	2	0.11	RBT	[23]
*Falcão* M et al.	2019	Brazil	Cattle	383	94	24.54	RBT	[34]
*Carvalho* L et al.	2022	Brazil	Cattle	964	2	0.21	RBT	[56]
*Gädicke* P et al.	2016	Chile	Cattle	400	0	0.00	RBT	[62]
*Tique* V et al.	2009	Colombia	Cattle	2,969	113	3.81	RBT	[48]
*Calderón-Rangel* A et al.	2013	Colombia	Cattle	246	7	2.85	IFAT	[49]
*Arena N*E et al.	2017	Colombia	Cattle	546	23	4.21	ELISA	[15]
*Cardenas* L et al.	2018	Colombia	Cattle	46,559,015	2,421,068	5.20	IFAT	[13]
*Motta-Delgado* P et al.	2020	Colombia	Cattles	959	31	3.23	RBT	[58]
*Moscoso-Gama* J et al.	2022	Colombia	Cattle	26,187	263	1.00	RBT	[53]
*Ramirez O* et al.	2022	Colombia	Cattle	656	43	6.55	RBT	[55]
*Raquel-Montes* M et al.	2022	Colombia	Buffaloes	144	5	3.47	RBT	[60]
*Hernández-Mora G* et al. (Cohort A)	2017	Costa Rica	Buffaloes	2,586	132	5.10	IFAT	[20]
*Hernández-Mora G* et al. (Cohort B)	2017	Costa Rica	Cattle	13,078	74	0.57	RBT	[33]
*Ruano M* et al.	2022	Cuba	Cattle	12,760	113	0.89	RBT	[54]
*Moyano J* et al.	2016	Ecuador	Cattle	48	0	0.00	ELISA	[69]
*Carbonero A* et al.	2017	Ecuador	Cattle	2,666	453	16.99	RBT	[45]
*Gioia G* et al.	2018	Ecuador	Cattle	410	62	15.12	RBT	[63]
*Rosero E* et al.	2018	Ecuador	Cattle	1,000	94	9.40	ELISA	[65]
*Changoluisa D* et al.	2019	Ecuador	Cattle	172	0	0.00	RBT	[57]
*Chikweto A* et al.	2013	Grenada and West Indies	Cattle	150	9	6.00	ELISA	[26]
*Zelaya M* et al.	2017	Guatemala	Cattle	36,602	3010	8.22	IFI	[39]
*Castilleja* Y et al.	2010	Mexico	Cattle	198	78	39.39	ELISA	[27]
*Rivera J* et al.	2010	Mexico	Cattle	993	723	72.81	ELISA	[37]
*Milián-Suazo F* et al.	2016	Mexico	Cattle	4,487	229	5.10	RBT	[68]
*Ojeda-Carrasco J* et al.	2016	Mexico	Cattle	331	0	0.00	ELISA	[73]
*Gutiérrez-Hernández J* et al. et al.	2020	Mexico	Cattle	5,382	8	0.15	RBT	[64]
*Rolon B*	2020	Paraguay	Cattle	58	0	0.00	RBT	[71]
*Valdivia* L et al.	2003	Peru	Cattle	385	0	0.00	RBT	[75]
*Huguet C* et al.	2005	Peru	Cattle	486	2	0.41	RBT	[59]
*Meza A* et al.	2010	Peru	Cattle	3,221	258	8.01	RBT	[46]
*Zavala I* et al.	2011	Peru	Cattle	5,430	1	0.02	IFAT	[41]
*Del Aguila W* et al.	2021	Peru	Cattle	286	0	0.00	RBT	[74]
*Ventocilla S* et al.	2004	Perú	Cattle	1,384	0	0.00	IFI	[47]
*D'Pool G* et al.	2004	Venezuela	Cattle	384	35	9.11	ELISA	[43]
*Castillo-Ojeda M* et al.	2011	Venezuela	Cattle	58	24	41.38	ELISA	[61]
*Rosales-Zambrano D* et al.	2015	Venezuela	Buffaloes	80	5	6.25	RBT	[72]

RBT: Rose Bengal test, 2MET: 2- mercaptoethanol test, ELISA: enzyme-linked immuno-sorbent assay, IFI: indirect immunofluorescence test (IFI), IFAT: immunofluorescence antibody test.

Our review included 65 studies published between 2002 and 2022, all of which were cross-sectional. A total of 46,883,177 animals were assessed, of which 2,433,382 were seropositive for Brucellosis. The studies were conducted in the following countries: Brazil (28 studies), Colombia (8 studies), Peru (6 studies), Ecuador (5 studies), Mexico (5 studies), Argentina (3 studies), Venezuela (3 studies), Costa Rica (2 studies), Chile (1 study), Cuba (1 study), Paraguay (1 study), Guatemala (1 study), Grenada and West Indies (1 study). Regarding the quality of the studies evaluated with the JBI checklist (Supplementary Table S3), we found 60 studies with a low risk of bias and five studies with a high risk of bias.

### 3.2 Prevalence of *Brucella* in bovines

The pooled seroprevalence of Brucellosis in cattle was 4.0% (95%CI: 3.0%–5.0%), with high heterogeneity (I^2^ ​= ​99.66%, p ​< ​0.001) (Table 2). In the analysis of subgroups according to countries (Table 2), no decrease in heterogeneity was found in any of the nations, Venezuela the country with the highest prevalence (16.0%, 95%CI: 3.0%–36.0%; p ​< ​0.001, I^2^ ​= ​99.56%). In the subgroup analysis according to regions (Table 2), there was also no decrease in heterogeneity in any of the areas: South America (3.0%,95%CI: 3.0%–4.0%; p ​< ​0.001, I^2^ ​= ​99.56%) vs Central America and the Caribbean (8.0%,95%CI: 3.0%–15.0%; p ​< ​0.001, I^2^ ​= ​99.85%). When stratified by species (Table 2), no decrease in heterogeneity was found in any of the subgroups: Cattle (3.0%, 95%CI: 3.0%–4.0%; p ​< ​0.001, I^2^ ​= ​99.7%) vs Buffaloes (7.0%, 95%CI: 4.0%–11.0%; p ​< ​0.001, I^2^ ​= ​98.12%). When evaluating the subgroup analyses according to year of publication, no decrease in heterogeneity was found in any of the subgroups: 2004–2010 (9.0%, 95%CI: 7.0%–10.0%; p ​< ​0.001, I^2^ ​= ​99.67%), 2011–2015 (3.0%, 95%CI: 2.0%–4.0%; p ​< ​0.001, I^2^ ​= ​98.03%) and 2016–2022 (4.0%, 95%CI: 3.0%–5.0%; p ​< ​0.001, I^2^ ​= ​99.91%). While in the subgroup analyzes according to diagnostic tests, no decrease in heterogeneity was found in any of the subgroups: RBT(4.0%, 95%CI: 3.0%–5.0%; p ​< ​0.001, I2 ​= ​99.21%), IFI (2.0%, 95%CI: 0.0%–8.0%; p ​< ​0.001, I^2^ ​= ​99.81%), IFAT(2.0%, 95%CI: 0.0%–7.0%; p ​< ​0.001, I^2^ ​= ​99.60%) and ELISA (13.0%, 95%CI: 10.0%–16.0%; p ​< ​0.001, I^2^ ​= ​99.63%). Finally, in the sensitivity analysis, after excluding those studies with a high risk of bias, no decrease in heterogeneity was found (4.0%, 95%CI: 3.0%–5.0%; p ​< ​0.001, I^2^ ​= ​99.68).

**Table 2:** Results of meta-analyses of prevalence.

Variable	Number of Studies	Pool Prevalence (%)	95%CI	n	I^2^	p
Overall seroprevalence	65	4.0	3.0–5.0	46,883,177	99.66%	**<0.001**
Central America and Caribbean Islands	10	8.0	3.0–15.0	76,567	99.85%	**<0.001**
Mexico	5	15.0	1.0–42.0	11,391	99.88%	**<0.001**
Costa Rica	2	1.0	1.0–1.0	15,664	–	**<0.001**
Grenada and West Indies	1	6.0	3.0–11.0	150	–	**<0.001**
Guatemala	1	8.0	8.0–9.0	36,602	–	**<0.001**
Cuba	1	1.0	1.0–1.0	12,760	–	**<0.001**
South America	55	3.0	3.0–4.0	46,806,610	99.56%	**<0.001**
Brazil	28	3.0	2.0–4.0	189,493	99.41%	**<0.001**
Argentina	3	12.0	0.0–39.0	9,927	–	**<0.001**
Perú	6	1.0	0.0–5.0	11192	98.35%	**<0.001**
Chile	1	0.0	0.0–1.0	400	–	**<0.001**
Venezuela	3	16.0	3.0–36.0	522	–	**<0.001**
Colombia	8	4.0	2.0–6.0	46,590,722	99.61%	**<0.001**
Ecuador	5	6.0	2.0–13.0	4,296	97.14%	**<0.001**
Paraguay	1	0.0	0.0–6.0	58	–	**<0.001**
Type of animals						
Cattle	55	3.0	3.0.4.0	46,868,688	99.7%	**<0.001**
Buffalos	10	7.0	4.0–11.0	14,489	98.12%	**<0.001**
Year of publication						
2004–2010	19	9.0	7.0–10.0	139817	99.67%	**<0.001**
2011–2015	12	3.0	2.0–4.0	28143	98.03%	**<0.001**
2016–2022	36	4.0	3.0–5.0	46742110	99.91%	**<0.001**
Diagnostic test						
Rose Bengal Test	43	4.0	3.0–5.0	217847	99.21%	**<0.001**
Indirect immunofluorescence test	3	2.0	0.0–8.0	46951	99.81%	**<0.001**
ELISA	13	13.0	10.0–16.0	34894	99.63%	**<0.001**
Immunofluorescence antibody test	5	2.0	0.0–7.0	46567413	99.60%	**<0.001**

## 4 Discussion

Brucellosis is still an infectious disease under surveillance in animals and humans. Infection due to *Brucella* species negatively impact animal and human health. Even though it has been subjected to extensive interventions and strategies for control, it is still prevalent in many countries worldwide, including most Latin American countries [1]. In addition to animal and human-associated factors, the environment appears to play a crucial role also, increasing, under appropriate circumstances, the transmission risk in endemic areas and ecological niches [76].

In the last two decades, the pooled prevalence of bovine brucellosis in Latin America showed a low but persistent seroprevalence of 4%. No significant variations were found in the period under study, except for 2010 and 2022, with variations that may be associated with ecological conditions of the region, among multiple factors. In Central America, the Caribbean islands subregion, including Mexico, a significantly higher seroprevalence was found (8%) compared to South America. The seroprevalence of Mexico would influence it, that yield 15%. Those seroprevalences would be affected by higher sensitivity of certain serological tests, that may yield false positives, leading to a possible higher seroprevalence than real. Then, it is possible that the seroprevalence of bovine brucellosis would be lower than observed. In any case, certainly control of bovine brucellosis should led to zero cases in most territories, and eventually to elimination and even eradication of those territories. Nevertheless, serological tests are the most used and important diagnostic tools for the massive screening and control programs of brucellosis, as has been stated in different studies [77].

In South America, most countries have control and eradication programs for bovine brucellosis. Among them, Uruguay and some Brazilian states show lower levels of prevalence over the last decade. In Chile, the Brucellosis Program of the Agricultural and Livestock Service seeks national eradication in the medium term. The south and north of the country are free while the central regions are not free [78].

On the other hand, in Colombia, the Program for the Prevention, Control and Eradication of Bovine Brucellosis is carried out. Its objective is to reduce the prevalence of the disease to levels that allow the eradication of the national territory [79]. Likewise, in Mexico, the State of Baja California Sur is recognized as free of brucellosis and Sonora is free of brucellosis; 29.16% of the national territory is recognized in the eradication phase [80].

In Argentina, the "Zone free of bovine brucellosis and tuberculosis", comprised the province of Tierra del Fuego, Antarctica and the South Atlantic Islands, declared by the SENASA (National Food Safety and Quality Service) Resolution [81]. On the other hand, in Peru has a program of control, but disease it is distributed throughout the country, especially in the dairy basins of Arequipa, Trujillo, Cajamarca and Lima, where the exploitation system is stabled or semi-stabled [82].

According to the WOAH Information Database, Mexico had the most reported outbreaks, 5,514 in 2014. Mexico is followed by China (2,138), Greece (1,268), and Brazil (1,142). Most of these outbreaks were related to *Brucella abortus*, the etiologic agent of bovine brucellosis [83]. As previously described, the risk of infection by *Brucella* in Mexico is associated with the consumption of unpasteurised dairy products, mainly fresh cheeses [84]. The interplay and importance of brucellosis in cattle are remarkable [85]. Brucellosis remains endemic in many regions, including Latin America, with morbidity much more prevalent in developing countries, such as Mexico.

In countries such as Brazil, recent analyses showed that vaccinating 90% of the replacement heifers aged 3–8 months of age offers the best cost-effectiveness in a vaccination program against bovine brucellosis if compared to vaccination rates of 70% and 80%. Moreover, regions with a higher prevalence of bovine brucellosis would experience significant economic advantages when implementing a vaccination strategy to control the disease. This financial analysis will allow decision-makers to plan more economically effective vaccination programs [86]. As Brazil has been successful with brucellosis vaccination, its seroprevalence is significantly lower than the observed in Mexico.

Previous studies suggested buffalos may be more resistant to infection than cattle [87], but we found a significantly higher seroprevalence in buffalos. In addition, some studies suggest such findings in their territories [88].

Finally, the large sample size of this meta-analysis, including more than 46 million animals, assessing the brucellosis in bovines in Latin America, calls for more studies and control to understand what is needed to improve and decrease more such seroprevalences (3–5%, 95%CI).

### 4.1 Limitations

This review must be interpreted considering its limitations. First, still, many studies are available for inclusion. It would be better to include as many more studies from other countries in Central America not represented in this study (e.g., El Salvador). Second, further information about the collected and sampled animals, particularly regarding their clinical findings and conditions, was unavailable in most studies; however, the data in this review permit a first synthesis of the frequency of infection due to *Brucella* spp in bovine, although the need to be more detailed for the last one. Among the important conditions are the vaccination status or the type of *Brucella* found, which were not reported in the included studies, and may generate false positives that overestimate the seroprevalence. Third, we used different serological tests to assess the seroprevalence, and certainly sensitivity and specificity varies among studies and testing methods, which is hard to directly adjust at a systematic review. All studies without exclusion of any diagnostic test were included. We performed a subgroup analysis where the differences between the diagnostic tests used in the included studies were evaluated. The included studies do not report the sensitivity and specificity values of the serological tests, so it is not possible to estimate the true prevalence. The use of these serological tests probably generates an overestimation of the prevalence due to the presence of false positives due to cross-reactions. Fourth, although some studies previously have suggested the influence of climatic factors in the prevalence of brucellosis [76], this can be directly assessed in this review, focused on the combined seroprevalence of disease. Ecological studies are necessary for that. There was no detailed information in most of the studies about the type of *Brucella* found in these animals. Finally, it would be interesting to assess risk factors including other type of studies at different individual and collective level.

## 5 Conclusions

Given their reported frequency, infection with *Brucella* spp in bovines is considered relevant in animals. Additional research is needed to elucidate multiple aspects of transmission, reinfection, coinfection, and many other ecological aspects of the disease, including the role of environmental issues related to their natural cycles. Further research should focus on developing studies that fully characterise and define the determinants of zoonotic spillover and their linkages to make operational contributions for risk assessment. Cross-species spillover is the defining characteristic of pathogens that transmit from vertebrate animals to humans, and zoonoses, as in bovine brucellosis. The public health burden imposed by zoonoses includes outbreaks of those pathogens.

The persistent, although low, seroprevalence of bovine brucellosis would be related to social, natural, and rising conditions. Bovine brucellosis should be zero in the countries. For instance, climate conditions favouring the survival and spread of pathogens in the environment; farms located in regions bordering forest areas of difficult access to veterinary services; extensive beef herds raised at pastures with different age and productive groups inter-mingled; and minimal concerns regarding hygiene practices and disease prevention measures. Bovine brucellosis has not been eliminated yet and needs to be reconsidered with new tools for prevention and control.

## Funding

From the Research Unit, Universidad Continental, Huancayo, Peru, for funding the 10.13039/100016205APC of this article. Study sponsors had no role in the study design, in the collection, analysis and interpretation of data, the writing of the manuscript, and the decision to submit the manuscript for publication.

## CRediT authorship contribution statement

**D. Katterine Bonilla-Aldana:** Conceptualization, Investigation, Formal analysis, Writing – original draft. **Adrián Esteban Trejos-Mendoza:** Conceptualization, Investigation, Formal analysis, Writing – original draft. **Soffia Pérez-Vargas:** Conceptualization, Investigation, Formal analysis, Writing – original draft. **Estefany Rivera-Casas:** Conceptualization, Investigation, Formal analysis, Writing – original draft. **Fausto Muñoz-Lara:** Conceptualization, Investigation, Formal analysis, Writing – original draft. **Lysien I. Zambrano:** Conceptualization, Investigation, Formal analysis, Writing – original draft. **Kovy Arteaga-Livias:** Conceptualization, Investigation, Formal analysis, Writing – original draft. **Juan R. Ulloque-Badaracco:** Methodology, Formal analysis, Writing – original draft. **Esteban A. Alarcon-Braga:** Methodology, Formal analysis, Writing – original draft. **Enrique A. Hernandez-Bustamante:** Methodology, Formal analysis, Writing – original draft. **Ali Al-kassab-Córdova:** Methodology, Writing – review & editing, Visualization, Supervision. **Vicente A. Benites-Zapata:** Methodology, Writing – review & editing, Visualization, Supervision. **Alfonso J. Rodriguez-Morales:** Conceptualization, Investigation, Writing – review & editing, Visualization, Supervision.

## Declaration of competing interest

The authors do not have conflicts of interest.
